# Endovascular treatment of a pseudoaneurysm in the right inferomedial genicular artery after arthroscopic anterior cruciate ligament reconstruction: a case report

**DOI:** 10.1093/bjrcr/uaaf014

**Published:** 2025-03-17

**Authors:** Miguel Barrio Piqueras, Cristobal Varela, Angel Javier Muñoz Vázquez, Antonio Martinez de la Cuesta

**Affiliations:** Department of Radiology, Clinica Universidad de Navarra, Navarra, Pamplona 31008, Spain; Department of Radiology, Clinica Universidad de los Andes, Santiago de Chile 293, Chile; Department of Traumatology, Clinica Universidad de Navarra, Pamplona, Navarra 31008, Spain; Department of Radiology, Clinica Universidad de Navarra, Navarra, Pamplona 31008, Spain

**Keywords:** pseudoaneurysm, inferomedial genicular artery, endovascular, anterior cruciate ligament

## Abstract

Rupture of the anterior cruciate ligament (ACL) is a common knee injury, and reconstruction via arthroscopy is popular for its safety and low complication rate, around 1%. Vascular injuries from this procedure are extremely rare, with an incidence of 0.003%-1%. Pseudoaneurysms following knee arthroscopy are even less frequent. This paper discusses a rare case of pseudoaneurysm after ACL reconstruction in the articular branch of the right inferomedial genicular artery (IMGA), originating from an anterior tibial artery with a high origin. A 47-year-old man with Von Willebrand disease developed a 24 mm pseudoaneurysm 30 days post-ACL reconstruction. CT-angiography showed the pseudoaneurysm near the tibial tunnel screw and an unusually high anterior tibial artery origin. Emergency angiography confirmed this, and embolization using Squid Peri 18 was successful, with no complications. The patient recovered well. Vascular injury is a rare knee arthroscopy complication, but early diagnosis and awareness of anatomical variations are essential. Endovascular treatment for iatrogenic pseudoaneurysms is safe and effective and facilitates rapid recovery.

## Background

Rupture of the anterior cruciate ligament (ACL) is one of the most prevalent knee injuries and consequently, the reconstruction of ACL is widely performed, commonly via arthroscopy. This technique is considered safe and has a low complication rate, with absolute risks associated with knee arthroscopy remaining minimal, at approximately 1%.^1^ Vascular injuries specific to this procedure are exceptionally rare, with studies indicating an incidence rate between 0.003% and 1%.^2^^,^^3^ Reports of pseudoaneurysm development after knee arthroscopy are significantly limited.

This paper presents a case of a pseudoaneurysm, developing after arthroscopic ACL reconstruction, in the articular branch of the right inferomedial genicular artery (IMGA), which itself arises from a right anterior tibial artery with a high origin.

## Case presentation

**Figure 1.**
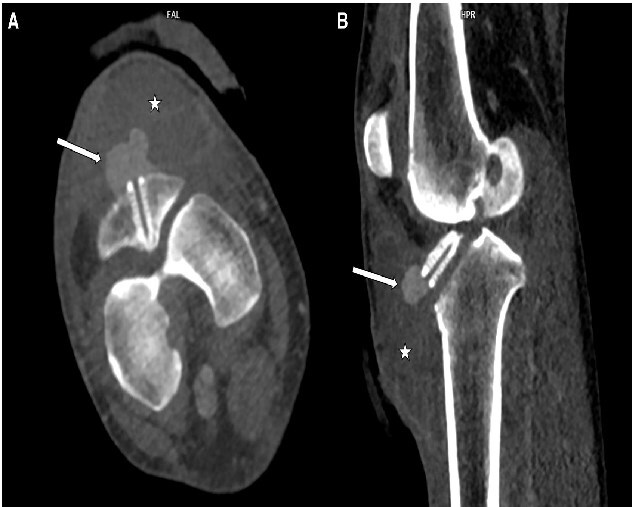
Axial (A) and sagittal (B) reconstruction of the CT angiography showing a haematoma (white star) and a hyperdense pseudonodular lesion in direct contact with the distal part of the tibial tunnel screw, compatible with a pseudoaneurysm.

A 47-year-old man, with a history of Von-Willebrand disease, was transferred to the Interventional Radiology department of our institution due to surgical wound dehiscence and suspected active bleeding 30 days after undergoing right ACL reconstruction. Upon admission, the patient was stable but showed a pulsatile swelling and signs of bleeding in the medial size of the treated knee. CT-angiography of the lower extremities revealed a significant haematoma associated with a 24 mm pseudoaneurysm, likely originating from the right IMGA that showed a downwards angulated origin. It was noted that the pseudoaneurysm was in close contact with the tibial tunnel screw (Figure 1). Additionally, an unusually high origin of the right anterior tibial artery (arising from the middle third of the right popliteal artery) was observed.

**Figure 2.**
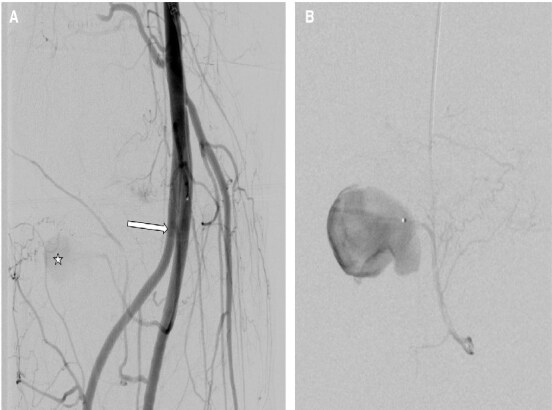
(A) Angiography was performed from the distal right femoral artery, confirming the presence of the pseudoaneurysm (star) and revealing that the origin of the inferomedial genicular artery (IMGA) was indeed in the previously described high-origin anterior tibial artery (arrow). (B) Ulterior catheterization of the IMGA with more detailed visualization of IMGA disposition and the pseudoaneurysm.

**Figure 3:**
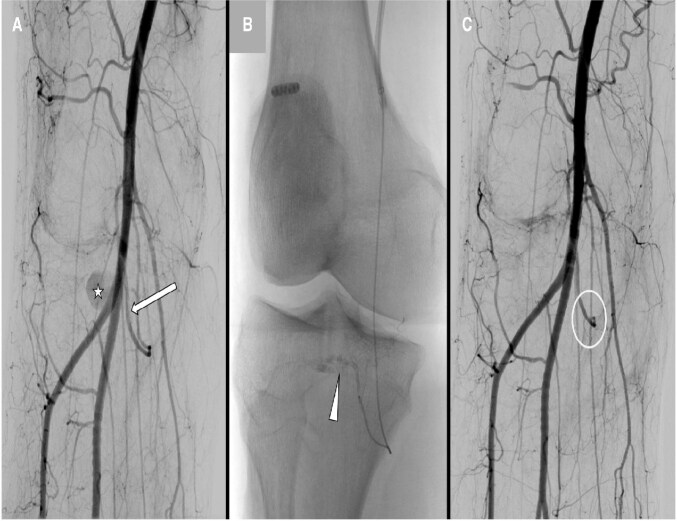
(A) Pre-embolization image before treatment. (B) Administration of the embolic agent from the proximal portion of the inferomedial genicular artery. (C) Comparative arteriography after embolization, showing successful occlusion of the aneurysm.

Emergency angiography was performed straightaway, under local anaesthesia and sedation, in the Interventional Radiology suite. The procedure involved retrograde puncture of the left common femoral artery with ulterior catheterization of the right common femoral artery, using a Cobra 5F catheter (Cordis Medical Corp., Miami Lakes, FL, USA), followed by the advancement of a 5F introducer sheath (Terumo Europe N.V., Leuven, Belgium) to the distal right femoral artery. The angiography confirmed the CT-angiography findings and also demonstrated an IMGA originating from the anterior tibial artery (Figure 2). Subsequently, the IMGA was catheterized with a 2.0 F Merit Pursue microcatheter (Merit Medical Ireland Ltd, Galway, Ireland), and the pseudoaneurysm was embolized with SquidPeri 18 (Balt, Gland, Switzerland) (Figure 3).

No complications were observed during the follow-up.

## Discussion

Pseudoaneurysms arise from a disruption in arterial wall continuity resulting from inflammation, trauma, or iatrogenic causes such as surgical procedures (in our case the tibial tunnel screw could have lacerated the arterial wall), percutaneous biopsy, or drainage. Under the influence of sustained arterial pressure, blood dissects into the tissues around the damaged artery and forms a perfused sac that communicates with the arterial lumen. The perfused sac is contained by the media or adventitia or simply by soft-tissue structures surrounding the injured vessel.^4^

Regarding knee procedures, the most commonly reported incidence of vascular injury and, consequently, pseudoaneurysms, occurs following total knee arthroplasty.^5^

As mentioned earlier, arthroscopy has a low complication rate. The absolute risk of complications associated with knee arthroscopy remains minimal, at around 1%.^1^ Vascular injuries are rare, with studies reporting an incidence between 0.003% and 1%.^2^^,^^3^

Pseudoaneurysms of the IMGA after arthroscopic ACL reconstruction are highly unusual and rarely described in the literature.^6^^,^^7^

Pseudoaneurysms can be treated with ultrasonography-guided compression, ultrasonography-guided thrombin, injection, open surgical ligation of the involved vessels, embolization by a microcatheter, or insertion of an endovascular stent.^8^

The traditional treatment for pseudoaneurysms has been surgical repair, but surgery has several drawbacks, including prolonged hospitalization, the requirement for general anaesthesia, and poor wound healing in patients with comorbidities. Additionally, surgery carries a higher risk of complications and mortality compared to interventional radiology techniques. Advances in technology have led to the increased use of interventional radiology, which is now the preferred treatment. However, surgery remains the gold standard for infected pseudoaneurysms, rapid expansion, ischaemia, neuropathy, and cases where percutaneous or endovascular treatments have failed.^9^

In our case, due to the presence of a significant haematoma, the risk of active bleeding and the possibility of an immediate endovascular access to the feeding vessel, the endovascular approach was performed with a successful outcome and a minimal recovery time for the patient.

Various embolic materials may be used for embolization of pseudoaneurysms, and the choice depends on various factors with no specific guidelines.

The decision to use a liquid agent in this case was due to the inability to advance the microcatheter beyond the proximal portion of the feeding artery due to its small calibre. By using a liquid agent that will flow across the pseudoaneurysm neck, distal and proximal embolization (sandwich technique) will be achieved, thereby preventing incomplete occlusion and development of collateral circulation.

A further point that makes this case particularly interesting is the presence of an unexpected anatomical variant consisting of an IMGA originating from the anterior tibial artery and not from the popliteal artery, together with a high origin itself of the anterior tibial artery from the middle third of the popliteal artery. To our knowledge, this variation has not been described before.^10^

## Conclusion

Vascular injury is an extremely rare complication of knee surgery; however, there should be awareness for its occurrence in order to establish an early diagnosis and treatment.

The possibility of anatomical variants should always be considered to ensure a faster and more effective therapeutic approach.

Endovascular treatment of iatrogenic pseudoaneurysms is a fast, feasible, and safe technique that yields excellent results and enables quick post-procedural recovery.

## Learning points

- Inferomedial genicular artery pseudoaneurysms are a rare complication of arthroscopic anterior cruciate ligament reconstruction.
- However, it is essential to consider this complication due to its high risk of haemorrhage. Awareness of this possibility allows for timely intervention and appropriate planning to manage potential vascular complications effectively.
- Endovascular treatment has proven to be a safe, straightforward, and effective approach for addressing this complication, significantly reducing the risks associated with open surgical management.
- Awareness of potential anatomical variations in knee arterial vascularization is crucial during procedural planning to avoid prolonged surgery times and reduce unnecessary radiation exposure.

## References

[1] Friberger Pajalic K , Turkiewicz A, Englund M. Update on the risks of complications after knee arthroscopy. BMC Musculoskelet Disord. 2018;19:179. 10.1186/s12891-018-2102-y 29859074 PMC5984803

[2] Neagoe RM , Bancu S, Muresan M, Sala D. Major vascular injuries complicating knee arthroscopy. Videosurgery Other Miniinvasive Tech. 2015;10:266-274. 10.5114/wiitm.2015.52559 PMC4520854 26240627

[3] Janssen RPA , Reijman M, Janssen DM, van Mourik JBA. Arterial complications, venous thromboembolism and deep venous thrombosis prophylaxis after anterior cruciate ligament reconstruction: a systematic review. World J Orthop. 2016;7:604-617. 10.5312/wjo.v7.i9.604 27672574 PMC5027016

[4] Saad NEA , Saad WEA, Davies MG, Waldman DL, Fultz PJ, Rubens DJ. Pseudoaneurysms and the role of minimally invasive techniques in their management. RadioGraphics. 2005; 25:S173-S189. 10.1148/rg.25si055503 16227490

[5] Wilson JS , Miranda A, Johnson BL, Shames ML, Back MR, Bandyk DF. Vascular injuries associated with elective orthopedic procedures. Ann Vasc Surg. 2003;17:641-644. 10.1007/s10016-003-0074-2 14534848

[6] Mello W , de Brito WE, Migon EZ, Borges A. Pseudoaneurysm of the medial inferior genicular artery after anterior cruciate ligament reconstruction. Arthroscopy. 2011;27:442-445. 10.1016/j.arthro.2010.10.015 21353173

[7] Filho ES , Isolani GR, Baracho FR, de Oliveira Franco APG, Ridder Bauer LA, Namba M. Pseudoaneurysm after arthroscopic procedure in the knee. Rev Bras Ortop (English Ed. 2015;50:131-135. 10.1016/j.rboe.2015.03.001 PMC4519561 26229905

[8] Farber A , Angle N, Avgerinos E, et al The society for vascular surgery clinical practice guidelines on popliteal artery aneurysms. J Vasc Surg. 2022;75:109S-120S. 10.1016/j.jvs.2021.04.040 34023430

[9] Sarioglu O , Capar AE, Belet U. Interventional treatment options in pseudoaneurysms: different techniques in different localizations. Polish J Radiol. 2019;84:e319-e327. 10.5114/pjr.2019.88021 PMC6798774 31636766

[10] Callese TE , Cusumano L, Redwood KD, et al Classification of genicular artery anatomic variants using intraoperative cone-beam computed tomography. Cardiovasc Intervent Radiol. 2023;46:628-634. 10.1007/s00270-023-03411-3 36949185 PMC10156764

